# Distinguishing Mood and Emotion: Implications for High-Performance Regulation

**DOI:** 10.3390/brainsci16020231

**Published:** 2026-02-14

**Authors:** Andrew M. Lane

**Affiliations:** Sport Physical Activity Research Centre, School of Health and Well-being, University of Wolverhampton, Walsall Campus, Walsall WS1 3BD, UK; a.m.lane2@wlv.ac.uk

**Keywords:** affect regulation, psychological adaptation, performance enhancement, mental health, self-awareness

## Abstract

**Highlights:**

**What are the main findings?**

- Clear differentiation between mood and emotion improves understanding of how affective states influence performance in high-pressure contexts.
- Accurate identification of the source and time-course of affective states enables more targeted and effective regulation strategies.

**What are the implications of the main findings?**

- Misclassifying moods as emotions (or vice versa) risks inappropriate regulation and inefficient use of self-regulatory resources.
- Applying structured, cause-aware frameworks supports more sustainable performance and wellbeing in demanding real-world environments.

**Abstract:**

Distinguishing mood from emotion has long posed challenges for psychology, with persistent definitional ambiguity limiting both theoretical precision and applied effectiveness. Our early work, identified duration and cause attribution as the most reliable markers differentiating short-lived, event-linked emotions from more diffuse, enduring moods. Researchers further advanced understanding by conceptualising emotions as feedback signals that support learning and adaptation, while the 4Rs model translated these insights into applied practice by embedding cause attribution within affect regulation. This paper integrates these conceptual, functional, and applied perspectives to demonstrate why accurate classification of affective states is a functional necessity in high-performance contexts. I propose that misclassifying moods and emotions may contribute to inefficient deployment of self-regulatory resources, whereas distinguishing states based on cause attribution may support more targeted and efficient regulation. Drawing on examples from sport, healthcare, performing arts, military operations, and corporate leadership, this paper synthesizes existing work to highlight the practical implications of the mood–emotion distinction for applied psychology.

## 1. Introduction

Understanding the distinctions and interactions between mood and emotion is central to explaining how people think, feel, and perform under pressure [1,2,3,4]. Classic accounts describe emotions as short-lived, object-focused, and action-oriented reactions to identifiable events [2,5,6], whereas moods are conceptualized as more diffuse, longer-lasting affective states that lack a clear or singular antecedent [7]. In everyday life, affective fluctuations typically shape well-being, motivation, and interpersonal functioning, with consequences that are often cumulative, diffuse, and reversible. By contrast, in high-performance environments such as surgery, military operations, competitive sport, and music performance, affective states unfold under conditions of time pressure, evaluative scrutiny, and high stakes, where even brief disruptions can have immediate and disproportionate consequences for attention, decision-making, technical execution, and resilience [8,9,10,11,12]. Despite the practical importance of these effects, the interaction between mood and emotion is rarely examined explicitly within the same study. This omission reflects a longstanding conceptual and methodological challenge: although moods and emotions clearly co-occur and interact in real-world performance, the field has lacked a practical framework for studying them together, with research typically privileging either transient emotional responses or broader mood states in isolation [2,13,14].

The conceptual separation of mood and emotion has been debated in psychology for decades, but one way its practical significance becomes readily apparent is in high-performance settings. Of the many high-performance environments, sport has been particularly influential in this regard, serving as a natural laboratory in which individuals repeatedly perform under time pressure, allowing fluctuations in affective states to be directly linked to observable behaviour and outcomes [15,16,17]. In such environments, affect is not simply experienced but actively regulated in the service of performance goals, making distinctions between transient emotional responses and more enduring mood states especially consequential [18]. Affect is used here as the term when feeling could be either a mood or an emotion rather than to introduce a third construct.

Despite longstanding definitional distinctions, early work in sport and performance psychology, including our own made limited effort to operationally differentiate mood from emotion. I refer to this trajectory explicitly to illustrate the conceptual development of the field rather than to privilege prior work. As researchers directly involved in the development of this framework, I recognise that my perspective is informed by sustained engagement with the reflective pathway that guided its formulation, including the practical judgments required when distinguishing mood from emotion in applied contexts. A meta-analysis in 2000 [19] treated affective states largely as a unified construct. While this advanced understanding by demonstrating robust links between affect and performance, it left important conceptual questions unresolved. In particular, ambiguity remained regarding whether studies assessed transient emotional episodes, enduring mood states, or an undefined combination of both. This imprecision increasingly constrained interpretation and the design of targeted interventions.

In response, a programme of research sought greater functional clarity. An analysis [2] of academic definitions and lay beliefs identified cause attribution, duration, and intensity as the most reliable differentiators: emotions were characterised as relatively intense, short-lived reactions to identifiable events, whereas moods were conceptualised as lower-intensity, longer-lasting states with less clearly defined causes. This clarification provided a foundation for more precise and actionable approaches to affect assessment and regulation.

Importantly, previous research [2] also engaged with alternative conceptualisations of affect, including dimensional and constructionist perspectives alongside categorical approaches. Constructionist accounts propose that emotions are constructed from core affective dimensions, typically valence and arousal combined with contextual interpretation and conceptual knowledge. Network approaches similarly conceptualise affective states as dynamic systems of interacting components rather than discrete psychological entities, while dimensional models emphasise continuous affective space rather than categorical boundaries. The present paper does not seek to adjudicate among these perspectives. Rather, building on earlier treatments, it adopts a pragmatic distinction based on duration and cause attribution because these features are particularly useful for applied regulation. In high-performance environments, regulatory decisions must often be made under time pressure and resource constraint. Distinguishing whether an affective state is short-lived and event-specific, or diffuse and longer-lasting, helps determine whether regulation should focus on resolving a situational trigger, restoring a broader mood state, or modulating excessive intensity. The duration–cause distinction therefore functions as a decision-oriented framework aligned with the applied goals of this paper.

Despite broad agreement at a conceptual level, definitional ambiguity persists in applied practice. This is embedded in how mood and emotion are measured and the language used to describe them. Terms such as ‘nervous’ or ‘tense’ are often used interchangeably to describe moods, emotions, or general affect within commonly used inventories, not because of measurement error, but because affective experiences unfold fluidly across timescales and contexts in real-world settings [2,13]. This ambiguity is particularly evident in high-performance environments. For example, a musician may experience “nerves” as an acute emotional spike immediately before performance, whereas individuals operating under sustained demand may describe a phenomenologically similar state as a gradual mood shift or persistent background tension [13,20]. Similarly, surgeons under acute stress show rapid surges in anxiety-like activation embedded within broader states of cognitive and physiological strain [12], while military personnel report longer-term mood erosion alongside sharp, situation-specific emotional episodes during operations [9,11]. Collectively, these examples illustrate that the lived experience of affect under pressure does not align neatly with traditional theoretical categories, rendering the simultaneous study of mood and emotion both necessary and methodologically demanding.

In high-pressure settings, individuals must regulate rapidly shifting internal states while maintaining precision, composure, and situational awareness [9,12]. Regulation, in this context, is best understood through a self-regulatory control framework [21,22,23]. From this perspective, regulation involves continuous monitoring of internal states relative to task-relevant reference values (e.g., optimal arousal, attentional focus, affective intensity). When a discrepancy is detected, such as anxiety exceeding functional levels [16], regulatory processes are engaged to reduce the gap between the current state and the desired state. Regulation therefore is not synonymous with suppression, but with feedback-guided adjustment aimed at maintaining effective functioning. Critically, the nature of the affective state being regulated determines the appropriateness of the regulatory response. Such regulation requires awareness of both current and ideal states. In high-pressure settings, misclassifying a short-lived emotional surge as a persistent mood, or interpreting accumulated mood drift as an acute emotional reaction, can lead to poorly matched regulation. A sudden spike in anxiety immediately before a procedure or performance typically reflects an event-linked emotional response and calls for rapid, situation-specific strategies (e.g., attentional refocusing or arousal modulation). In contrast, mood states shaped by fatigue, workload, or environmental strain reflect longer-term deviations in organismic condition and require sustained regulatory adjustments such as recovery, pacing, or workload modification [20,24].

Evidence from surgical stress, military resilience, and music performance anxiety demonstrates that ineffective regulation often arising from misclassification of affective states contributes to attentional tunnelling, impaired decision-making, and degraded performance under pressure [11,12,13]. Within a control-theoretic framework, these failures reflect breakdowns in accurate state monitoring or inappropriate selection of corrective actions [21,23,25]. Differentiating mood from emotion is therefore not a semantic exercise but a functional necessity in environments where regulatory resources are limited and small errors can have disproportionate consequences.

This article revisits the mood–emotion distinction by integrating conceptual, functional, and applied perspectives to show why the relationship between these states matters in high-performance settings. I propose that accurate cause attribution plays an important role in effective self-regulation. Misclassifying a persistent mood as a fleeting emotion or interpreting an acute emotional response as a stable mood state, may contribute to the selection of suboptimal regulatory strategies and inefficient use of limited psychological resources [2,3]. The present article adopts a conceptual narrative review approach. Its purpose is not to provide a systematic or exhaustive synthesis of all empirical studies on mood and emotion, but to integrate foundational theoretical perspectives with selected empirical evidence in order to clarify the functional distinction between these constructs. Literature has been drawn purposively from influential theoretical accounts and applied performance research to support conceptual integration and translational interpretation. The aim is theoretical refinement and applied clarity rather than comprehensive literature aggregation. Drawing on examples from sport, healthcare, performing arts, military operations, and corporate leadership, I illustrate how accurate classification shapes regulatory choices, recovery decisions, and sustained performance under pressure [9,12,20]. Rather than proposing a new model, this paper synthesises existing work, including the empirically grounded distinctions articulated previously [2], the functional account of emotion as feedback for goal regulation described previously [3], and the applied structure offered by the 4Rs model for monitoring and responding to affective states [20]. Taken together, these frameworks provide a coherent lens for understanding how mood and emotion interact to shape performance, regulation, and wellbeing in demanding environments.

## 2. Early Work Distinguishing Mood from Emotion

As suggested previously, sport provides a particularly instructive starting point for examining the mood–emotion distinction because applied sport psychology was among the earliest fields to embed affective measurement directly into routine performance practice [17]. Coaches and practitioners sought tools that could be administered repeatedly, interpreted quickly, and linked to training and competition outcomes. Within this applied context, early attempts to distinguish mood from emotion were shaped largely by the measurement instruments adopted rather than by prior conceptual clarity. Instruments such as the Profile of Mood States [26] and the Positive and Negative Affect Schedule [27] became foundational because they offered practical, standardized methods for quantifying affective states. Their prominence, however, reflected a broader pattern in applied psychology: empirical progress was often driven by the availability of usable measures before robust theoretical agreement on core constructs had been established [28,29]. As a consequence, mood was frequently assessed as an umbrella construct, with limited specification of what it represented, how stable it was, or how it differed from emotion. When instruments such as the POMS [26] were used, mood and emotion were often treated interchangeably, leaving conceptual boundaries implicit and constraining interpretability and the development of targeted regulation strategies.

As applied sport psychology evolved from a predominantly practice-driven discipline into a cumulative science–practice field, the limitations of conceptually imprecise constructs became increasingly apparent, prompting calls for greater theoretical clarity and definitional precision [30,31,32,33]. Early measurement-led approaches were instrumental in demonstrating robust relationships between affective states and performance [34] but they also exposed a broader scientific requirement: for findings to accumulate coherently and inform theory, core constructs must be defined with sufficient precision to support comparison, interpretation, and replication. It was within this context that Lane and Terry [1] offered one of the earliest sport-specific attempts to conceptualize mood in theoretically grounded terms. Rather than treating mood as a unitary or residual category, they described it as a configuration of multiple feeling states that vary in duration and intensity, highlighting its diffuse, multi-component nature and its role as a background affective context for behavior. This account explicitly distinguished mood from discrete emotional episodes, conceptualized as shorter-lived and more tightly linked to identifiable triggers. Importantly, the framework did not resolve the mood–emotion distinction but clarified why it mattered. By acknowledging that moods and emotions share phenomenological features, unfold across overlapping timescales, and interact dynamically, it revealed the limits of existing measurement approaches and underscored the need for further conceptual and methodological development as the field progressed.

Beedie et al. [2] undertook a systematic effort to clarify the distinction between mood and emotion at a point when the field faced a clear conceptual bottleneck. They recognised that, despite decades of theoretical discussion, existing definitions lacked the coherence and operational specificity required for consistent application in applied settings. Academic accounts offered partially overlapping and sometimes contradictory definitions, while practitioners and performers frequently reported using the terms interchangeably in everyday language. To address this disconnection, Beedie et al. conducted a comprehensive review of more than 100 academic definitions and complemented this analysis with written responses from 106 participants describing how they personally distinguished mood from emotion. By examining both scholarly theory and lay understanding, the study assessed whether intuitive distinctions aligned with, refined, or challenged academic conceptualizations, arguably, an essential step in developing constructs that are not only theoretically defensible but also meaningful and usable in applied environments. Their findings revealed strong convergence around two core differentiators: duration and cause attribution. Emotions were typically characterized as short-lived responses to identifiable events, whereas moods were described as longer-lasting, lower-intensity states with less clearly defined causes—findings consistent with existing definitions.

Beedie et al. [2] also highlighted the practical difficulty of distinguishing mood and emotion in lived experience. For example, an athlete may experience sustained, high-intensity irritability driven by accumulated fatigue, a mood, while also encountering an equally intense surge of anger following an officiating decision, classified as emotion. This experiential ambiguity exposes a key limitation of relying on phenomenology alone to differentiate affective states. When intensity, valence, or subjective feel are insufficient discriminators, conceptual progress requires a shift away from how affect feels toward what affect does. At this juncture [3], advancing a functional interpretation of affect by reframing emotions not as direct causes of behavior, but as informational signals that support learning, anticipation, and reflection is a pivotal contribution. Baumeister et al. [3] offered a central insight to redirect the question from what emotions make people do to what emotions communicate about the world and the self.

Within this framework [3], emotions function as rapid, situation-specific feedback signals that highlight immediate opportunities, errors, or threats, whereas moods operate as broader, integrative indicators of internal conditions such as fatigue, recovery status, or motivational readiness. This functional distinction provides a principled basis for differentiating moods and emotions even when they are similar in intensity or valence. Crucially, because moods and emotions convey different kinds of information, confusing one for the other carries regulatory costs [4]. Treating a mood-related decline in energy as an emotion about the task or interpreting an acute emotional response as a generalized mood state, can result in misdirected self-regulation and inefficient preparation. These risks are particularly salient in high-pressure performance environments, where decisions about effort allocation, attentional focus, and recovery must be made under pressure and with limited psychological resources [9,20].

### 2.1. Baumeister’s Functional Model of Emotion

Baumeister et al. [3] offered a major reappraisal of how emotions support human functioning by shifting attention away from emotions as direct causes of behavior toward their role as informational signals. In this account, emotions provide feedback that supports learning, anticipation, and behavioral adjustment through reflection and self-regulation. Rather than mechanically triggering action, emotional responses signal whether current behavior is aligned with goals, values, or environmental demands, thereby shaping future decision-making. This functional interpretation provides a critical bridge between conceptual distinctions and applied regulation. If emotions and moods convey different kinds of information with emotions highlighting situation-specific feedback and moods reflecting broader internal conditions, then accurate identification of the experienced state becomes a practical necessity rather than a semantic concern. Misidentifying the informational source risks responding to the wrong signal, undermining effective regulation in high-performance contexts where timely and proportionate responses are essential.

Baumeister’s functional framework identifies three interlocking roles through which emotions support adaptive behavior [3]:

- Feedback. Emotions provide rapid evaluative feedback about recent actions. Feelings such as pride reinforce effective behavior, disappointment signals errors, and guilt indicates violations of personal, social, or professional standards. In high-pressure settings, this immediate affective feedback enables performers to adjust behavior more efficiently than deliberate cognitive analysis alone, particularly when time and attentional resources are constrained [9,12].
- Anticipation. Anticipated emotions shape behavior prospectively. Anticipated regret can motivate more thorough preparation, anticipated anxiety may prompt rehearsal of complex or high-risk tasks, and anticipated pride can support persistence and effort investment. Through this anticipatory function, emotions influence decision-making well in advance of performance, shaping how individuals allocate effort, manage risk, and prepare for demanding situations.
- Reflection. Emotional experiences are encoded and integrated into long-term memory, strengthening learning over time. Positive emotions reinforce successful strategies, whereas negative emotions discourage repetition of ineffective or harmful actions. This reflective function underpins structured learning practices such as coaching debriefs, after-action reviews, and professional supervision across performance environments, where emotional responses help consolidate lessons from experience and guide future behavior [11].

Baumeister et al. [3] also distinguished between automatic affective reactions and more conscious emotional processes. Automatic reactions involve rapid shifts in valence that support immediate readiness, orient attention, and mobilize action in response to perceived opportunities or threats. By contrast, conscious emotions entail more deliberate appraisal and self-evaluation, enabling individuals to interpret events in relation to goals, values, and standards. This dual-process structure is especially relevant in high-performance settings, where performers must manage fast, automatic stress responses alongside slower, reflective emotional processes to maintain effective regulation under pressure [9,12,20]. However, Baumeister et al. do not tackle the issue of mood and emotion distinctions head on or attempt to do that and build it into a practical framework such as the 4Rs model [4].

### 2.2. The 4Rs as a Bridge Between Theory and Applied Practice

Building on conceptual advances in distinguishing mood from emotion, the 4Rs model [4] (Recognise, Restore or Resolve, Regulate) offers a translational framework designed for use in applied performance environments. The central strength of the model lies in its procedural integration of cause attribution and temporal perspective into affect regulation, converting theoretical distinctions into actionable decisions under real-world constraints.

The first component, Recognise, involves more than noticing an affective state. It requires performers to identify the likely source and time-course of that state, specifically, whether it reflects a short-lived emotional response to a discrete event or a more sustained mood shaped by cumulative conditions. This explicit focus on cause attribution directly addresses the experiential ambiguity frequently reported in applied settings [2] and aligns with functional accounts of affect in which different affective states convey different forms of information [3]. Accurate recognition is therefore foundational, as it determines the appropriateness of subsequent regulatory action.

Following recognition, the model differentiates between Resolve and Restore, reflecting the fundamentally different regulatory demands of emotions and moods. Emotions, which arise rapidly in response to identifiable situational triggers, are typically amenable to resolution through short-acting, context-specific strategies such as attentional refocusing, cognitive reframing, or arousal modulation. Moods, by contrast, emerge gradually and reflect broader organismic conditions; they therefore require restorative responses that address underlying contributors such as fatigue, sleep disruption, workload, recovery practices, or social and environmental strain [4,20]. This distinction helps ensure that regulatory effort is proportional to the informational content of the affective signal. The Regulate component refers to the implementation and ongoing adjustment of strategies selected through recognition and classification. Regulation in the 4Rs is not a single act but a dynamic process, involving monitoring whether the chosen response is reducing the discrepancy between the current affective state and task-relevant demands [20,21,22,23]. Importantly, regulation remains contingent on continued appraisal of effectiveness. If an intervention fails to shift the affective state as expected, performers are implicitly returned to recognition and reclassification, refining their understanding of cause and time-course. In this way, reflection is embedded within the regulatory loop, rather than treated as a discrete final stage.

This embedded feedback process is particularly important in preventing common regulatory errors. Treating moods as if they were emotions, by repeatedly applying short-term psychological techniques to problems rooted in cumulative fatigue or overload often leads to frustration, ineffective regulation, and unnecessary depletion of psychological resources [2,3]. The 4Rs mitigate this risk by ensuring that regulation is continuously informed by updated recognition of the affective state.

Within this framework, moods serve a critical monitoring function in high-performance environments. Whereas emotions signal immediate situational demands, moods act as lower-frequency indicators of internal condition, reflecting accumulated load, recovery status, and resilience over time [2,3]. Persistent low energy, irritability, or emotional flatness in athletes, soldiers, clinicians, or musicians typically signals underlying physiological or cognitive strain rather than an event-specific emotional response [11,20]. Evidence from sleep, academic performance, and military contexts indicates that such mood drift often precedes observable performance decline, biasing attention, judgment, and decision-making capacity [9,14].

By embedding mood monitoring within an iterative recognition–regulation cycle, the 4Rs support early detection of overload before performance errors or wellbeing costs become apparent. This is especially valuable in contexts where objective performance indicators lag behind internal strain, such as intensive training phases, extended deployments, or prolonged periods of high clinical demand [9,20]. Overall, the 4Rs model provides a structured yet flexible bridge between theory and practice. Rather than requiring performers to resolve abstract conceptual distinctions in advance, the model operationalizes mood–emotion differentiation procedurally, allowing interpretation and regulation to unfold dynamically within the constraints of real-world performance environments. In doing so, the 4Rs function not as a competing theory of affect, but as a translational framework that connects established conceptual insights with the moment-to-moment demands of high-performance regulation.

What remains is to show how this translational logic operates in practice. Because affective demands, temporal pressures, and performance consequences vary substantially across domains, the utility of the 4Rs is best illustrated through applied case studies. The examples that follow demonstrate how recognizing, restoring or resolving, and regulating affective states are enacted in context, highlighting both common regulatory principles and domain-specific adaptations.

## 3. Applied Contexts

The performance contexts used as examples in this paper, sport, healthcare and surgery, performing arts, and military operations, were selected deliberately rather than exhaustively. These domains differ substantially in surface characteristics, including task structure, time horizons, professional cultures, norms surrounding emotional expression, and the consequences of error. Sport involves repeated performance cycles and explicit affect monitoring; surgery demands sustained precision under acute time pressure; performing arts combine expressive demands with evaluative threat; and military operations require prolonged functioning under chronic physical and psychological load.

**Figure 1:**
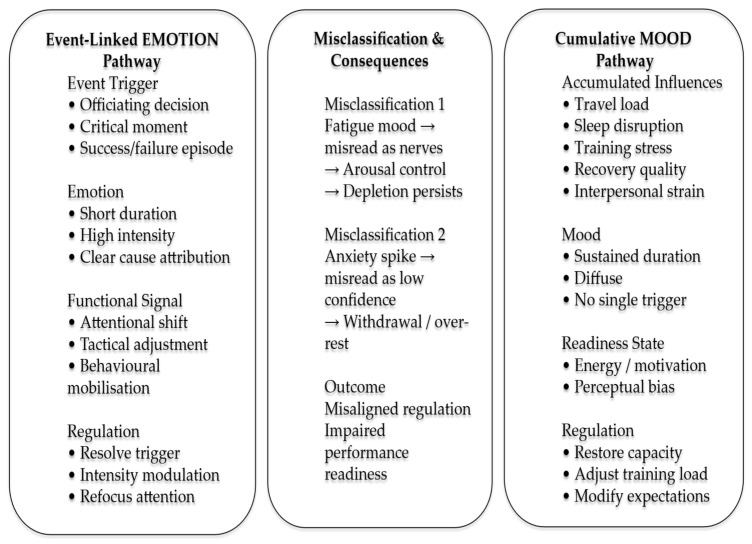
Conceptual model illustrating the distinction between event-linked emotions and cumulative mood states in sport performance contexts. Emotions are short-lived responses to identifiable triggers and require resolution-focused regulation. Moods reflect sustained readiness states arising from cumulative influences and require restorative strategies. Misclassification of these states leads to regulatory mismatch and impaired performance readiness.

Despite these differences, each context presents the same underlying regulatory challenge: individuals must interpret affective signals accurately under constraint and select proportionate regulation strategies using finite cognitive and emotional resources. The recurrence of the mood–emotion misclassification problem across such divergent environments supports the argument that the distinction is not domain-specific but structural. These examples therefore serve not as representative cases, but as theoretically informative contrasts that demonstrate the generality of cause-aware classification as a foundation for effective regulation. We present a conceptual model to illustrate the distinction between event linked emotions and cumulative mood states in contexts in Figure 1, using the example of sport.

### 3.1. Sport

Athletes operate in environments characterized by evaluative scrutiny, physical fatigue, unpredictable events, and high-performance stakes. Within these contexts, emotional spikes such as anger following an officiating decision, anxiety before a critical moment, or excitement after a successful action and function as short-lived, event-linked signals that guide immediate tactical, attentional, and behavioral adjustments [2,3] (see Figure 1). Mood states, by contrast, reflect the cumulative influence of broader factors such as travel demands, training load, sleep disruption, recovery quality, and interpersonal dynamics, shaping readiness and perception across time rather than in response to a single event [9,20].

Misclassification of these states is common in applied sport settings. An athlete may interpret several days of irritability and low energy, a mood state indicative of accumulated fatigue or inadequate recovery, as pre-competition nerves, prompting reliance on short-term arousal management strategies that do little to address the underlying cause [2,4]. Conversely, a brief surge of anxiety immediately before performance may be interpreted as a generalized loss of confidence or readiness, leading to unnecessary rest, withdrawal, or overly conservative pacing strategies that are disproportionate to the situational demand. In both cases, performance readiness is compromised because the regulatory response does not align with the source, duration, or functional meaning of the affective signal. These examples illustrate why accurate distinction between mood and emotion is essential for selecting regulation strategies that protect both immediate performance and longer-term sustainability.

### 3.2. Healthcare and Surgical Performance

Surgical and clinical environments place exceptional demands on precise motor control, sustained attention, and real-time decision-making under conditions of uncertainty and time pressure. Within these contexts, emotions arise rapidly in response to intraoperative events such as unexpected bleeding, equipment malfunction, or procedural deviation, functioning as situation-specific signals that mobilize attention, prioritize action, and support rapid problem-solving [3,9]. Mood states, by contrast, reflect accumulated influences including sleep quality, workload intensity, shift patterns, and interpersonal dynamics within clinical teams, shaping baseline readiness and cognitive efficiency across time rather than in response to a single event [20].

Evidence from surgical performance research indicates that clinicians often experience background tension, fatigue, or irritability that persists across days or weeks, yet describe these states as momentary “stress” during procedures, obscuring the mood-level vulnerabilities that undermine concentration, judgement, and error detection [12]. Conversely, brief emotional spikes during challenging moments may be misinterpreted as signs of more generalized decline in wellbeing, prompting overly broad interventions or unnecessary withdrawal from practice that are disproportionate to the situational demand. In both cases, performance risk increases because the regulatory response fails to align with the underlying source of the affective signal. Effective performance in healthcare settings therefore depends on accurate differentiation between situational emotional reactions and mood-level vulnerabilities, enabling regulation strategies that preserve precision, decision quality, and patient safety under sustained demand.

### 3.3. Performing Arts

Performing artists, particularly musicians, operate in emotionally charged environments characterized by evaluative threat, audience scrutiny, and high expressive demands. In these contexts, emotions such as pre-performance anxiety or excitement typically emerge immediately before performance and are closely linked to situational cues, including audience presence and imminent evaluation [3,13]. Mood states, by contrast, reflect broader and more enduring patterns of exhaustion, worry, or background tension arising from accumulated practice demands, touring schedules, sleep disruption, and ongoing personal or professional pressures.

Research on music performance anxiety indicates that performers frequently conflate mood states arising from poor sleep, repetitive strain, or prolonged stress with acute emotional activation associated with the performance moment itself [13]. As a result, musicians may rely primarily on last-minute emotion-regulation strategies, such as breathing routines or centering techniques, while neglecting mood-level contributors that require longer-term intervention, including recovery deficits, workload imbalance, or persistent cognitive worry. Distinguishing between mood and emotion therefore enables more targeted preparation, supports healthier regulation choices, and promotes sustainable artistic performance across repeated evaluative demands.

### 3.4. Military Operations

Operational military environments demand sustained vigilance, rapid threat appraisal, and effective functioning under extreme physical and psychological load. Within these contexts, emotions such as fear, anger, or relief arise rapidly in response to specific events—such as contact with adversaries, near misses, or sudden shifts in threat level—and function as situation-specific signals that support readiness, prioritization, and survival-oriented behaviour [9]. Mood states, by contrast, reflect cumulative influences including sleep deprivation, environmental hardship, unit cohesion, and prolonged operational tempo, shaping baseline motivation, attentional capacity, and resilience across time rather than in response to discrete incidents [11,20]. Research on military resilience and cognitive functioning under sustained stress highlights frequent misinterpretation of mood states as emotional volatility. Prolonged irritability, reduced motivation, or emotional blunting driven by exhaustion and extended operational demand are often labelled generically as “stress,” leading personnel to rely on short-term coping strategies that fail to address underlying physiological and cognitive depletion [11]. Conversely, transient emotional reactions to acute events may be interpreted as indicators of more enduring psychological decline, prompting responses that are disproportionate to the situational demand. In both cases, operational effectiveness is compromised because regulatory efforts are misaligned with the source and time-course of the affective signal. Clear differentiation between mood and emotion is therefore essential not only for individual performance and wellbeing, but also for effective leadership judgement, appropriate support, and sustained operational capability in high-risk environments.

### 3.5. Corporate Leadership and Executive Performance

Leadership roles involve sustained cognitive load, accountability, interpersonal demands, and rapid decision-making under conditions of uncertainty. In these contexts, emotions typically arise in response to acute events such as interpersonal conflict, critical feedback, unexpected failure, or emerging opportunity, functioning as situation-specific signals that orient attention and guide immediate judgement [3]. Mood states, by contrast, evolve through cumulative influences including workload intensity, organizational climate, sleep patterns, and prolonged pressure, shaping baseline motivation, tolerance for ambiguity, and decision quality across time [4,9].

Misclassification of these affective states is common in leadership settings. A diffuse mood characterized by fatigue, irritability, or disengagement may be attributed to a single emotionally demanding meeting, prompting surface-level regulatory responses such as cognitive reframing or motivational self-talk that fail to address underlying structural contributors, including workload imbalance, inadequate recovery, or persistent organizational strain [20]. Conversely, an acute emotional reaction to a high-stakes decision may be misinterpreted as evidence of longer-term decline in confidence or capability, leading to unnecessary strategic shifts, overcorrection, or destabilizing changes in team dynamics. Accurate classification of mood and emotion therefore supports more proportionate regulation, preserves cognitive clarity under uncertainty, and enables leaders to make decisions aligned with both immediate situational demands and longer-term organizational goals.

### 3.6. Convergence Across Domains

Across these five performance environments, a consistent pattern emerges. Emotions function as rapid, situation-specific signals that support immediate action and adjustment, whereas moods convey broader information about resource availability, readiness, and vulnerability that unfolds over time. When these signals are misclassified, regulation becomes inefficient: performers apply strategies that do not match the underlying cause, expend cognitive and emotional resources unnecessarily, and compromise performance. The applied illustrations demonstrate that this problem is not domain-specific but structural. Frameworks such as the 4Rs address this challenge by embedding cause attribution at the centre of decision-making, enabling regulatory strategies to be allocated in proportion to the source and time-course of the affective state. Together, these examples show that although the subjective experience of affect varies across contexts, the principles governing mood–emotion interactions are remarkably consistent across high-performance domains.

## 4. Future Research: Testing Cause-Aware Classification

Although the conceptual and functional rationale for distinguishing moods from emotions is now well established, empirical evaluation of cause-aware classification remains limited. The central priority for future research is therefore not further definitional refinement, but systematic testing of whether identifying the source and time-course of affective states improves regulation, performance, and wellbeing in applied settings.

Several methodological approaches offer feasible and rigorous pathways for addressing this question. Ecological momentary assessment can capture affective states across multiple timescales and contexts, allowing examination of whether cause attribution improves real-time regulatory accuracy. Experimental and field-based designs can independently induce acute emotional responses or more diffuse mood states, enabling direct tests of whether structured classification, such as that provided by the 4Rs, leads to superior regulatory outcomes compared with unguided or intuitive responses. Brief training or micro-intervention studies may assess whether even minimal instruction in cause-aware classification enhances perceived control, recovery efficiency, or decision quality. Longer-term observational designs can examine how mood–emotion patterns evolve under sustained demand and whether classification accuracy predicts resilience over time. Qualitative approaches may further illuminate how performers naturally interpret affective signals and whether structured frameworks alter sense-making in practice.

Taken together, these approaches provide a coherent and actionable agenda for evaluating whether cause-aware classification delivers measurable benefits across performance domains. The key question for future research is therefore empirical rather than conceptual: does embedding cause attribution into emotion regulation improve outcomes where it matters most?

This review demonstrates both continuity and progression in understanding the distinction between moods and emotions. Across theoretical, functional, and applied perspectives, duration and cause attribution emerge as the most reliable markers for differentiating the two constructs [2]. Baumeister et al.’s [3] functional model clarifies why this distinction matters by framing emotions as rapid, event-linked signals that support feedback, anticipation, and learning, while moods operate as broader indicators of underlying organismic state. The 4Rs model translates these insights into applied practice by embedding cause attribution within a structured regulatory sequence that can be implemented under real-world performance constraints [4].

Across sport, healthcare, performing arts, military operations, and leadership contexts, a consistent pattern is evident. Emotional states are short-lived and situation-specific, typically requiring rapid, tactical regulation strategies, whereas mood states are more diffuse and enduring, often signaling cumulative load, unresolved demands, or resource depletion and therefore requiring longer-term, systemic intervention. When these states are misclassified, regulation becomes inefficient: cognitive and emotional resources are expended unnecessarily, underlying drivers remain unaddressed, and performance under pressure is compromised.

Viewed through a functional control perspective, this distinction reflects differences in the source and persistence of regulatory error. Emotions signal immediate discrepancies linked to specific events, whereas moods reflect more sustained mismatches associated with accumulated strain or unmet demands. Accurate classification therefore enables performers to locate the source of disruption more precisely and select regulatory strategies proportionate to both the origin and time-course of the affective signal. The practical value of this distinction lies not in semantic precision, but in enabling more efficient and sustainable regulation.

These insights have clear implications for both measurement and practice. The timing and context of affective assessment are critical for interpretation, particularly when instruments designed to capture mood are administered in emotionally charged moments. Incorporating prompts that encourage reflection on cause and duration can strengthen construct validity and support more accurate differentiation in applied settings. In practice, explicitly teaching the mood–emotion distinction and embedding frameworks such as the 4Rs within coaching, supervision, clinical training, military preparation, and leadership development offers a pragmatic route to improving regulation accuracy, resilience, and performance sustainability.

## 5. Conclusions

The distinction between mood and emotion has challenged psychology for decades. The synthesis presented in this article demonstrates that the field now possesses the conceptual, functional, and applied tools required for meaningful clarity. Duration and cause attribution provide a stable definitional foundation for differentiating affective states. Baumeister et al.’s functional account explains why this distinction matters, framing emotions as rapid, event-linked signals that guide feedback, anticipation, and reflection, and moods as broader indicators of underlying organismic condition. The 4Rs framework builds on these insights by offering a practical, stepwise method for identifying and regulating affective states within real-world performance environments.

The central implication is that accurate, cause-aware classification is not an academic subtlety but a functional necessity. When moods are confused with emotions, regulation strategies are misapplied, finite self-regulatory resources are wasted, and vulnerability to performance error increases. When affective states are correctly classified, regulation becomes more targeted, efficient, and sustainable. This distinction is particularly critical in high-pressure environments where cognitive bandwidth is limited and the consequences of error are substantial.

Although empirical work integrating mood and emotion assessment remains limited, the logic underpinning cause-aware classification is now well specified. The challenge ahead is therefore empirical rather than conceptual. The tools exist; the next step is to test them systematically. Doing so offers psychology a route toward a more integrated, resource-efficient approach to affect regulation—one capable of supporting performance and wellbeing across sport, healthcare, performing arts, military operations, and leadership contexts.

## Data Availability

No new data were created or analyzed in this study. Data sharing is not applicable to this article.
